# The rebellious man: Next-of-kin accounts of the death of a male relative on antiretroviral therapy in sub-Saharan Africa

**DOI:** 10.1080/17441692.2019.1571092

**Published:** 2019-01-28

**Authors:** Morten Skovdal, Robert Ssekubugu, Constance Nyamukapa, Janet Seeley, Jenny Renju, Joyce Wamoyi, Mosa Moshabela, Kenneth Ondenge, Alison Wringe, Simon Gregson, Basia Zaba

**Affiliations:** a Department of Public Health, University of Copenhagen, Copenhagen, Denmark; b Rakai Health Sciences Program, Kampala, Uganda; c Biomedical Research and Training Institute, Harare, Zimbabwe; d Department for Infectious Disease Epidemiology, Imperial College London, London, UK; e London School of Hygiene and Tropic Medicine, London, UK; f Medical Research Council/Uganda Virus Research Institute, Entebbe, Uganda; g African Health Research Institute, Durban, South Africa; h Malawi Epidemiology and Intervention Research Unit, Karonga, Malawi; i National Institute for Medical Research, Mwanza Research Centre, Mwanza, Tanzania; j University of KwaZulu-Natal, Durban, South Africa; k Kenya Medical Research Institute, Kisumu, Kenya

**Keywords:** Antiretroviral treatment, HIV, illness-narratives, social autopsy, sub-Saharan Africa

## Abstract

The HIV response is hampered by many obstacles to progression along the HIV care cascade, with men, in particular, experiencing different forms of disruption. One group of men, whose stories remain untold, are those who have succumbed to HIV-related illness. In this paper, we explore how next-of-kin account for the death of a male relative. We conducted 26 qualitative after-death interviews with family members of male PLHIV who had recently died from HIV in health and demographic surveillance sites in Malawi, Tanzania, Kenya, Uganda, Zimbabwe and South Africa. The next-of-kin expressed frustration about the defiance of their male relative to disclose his HIV status and ask for support, and attributed this to shame, fear and a lack of self-acceptance of HIV diagnosis. Next-of-kin painted a picture of their male relative as rebellious. Some claimed that their deceased relative deliberately ignored instructions received by the health worker. Others described their male relatives as unable to maintain caring relationships that would avail day-to-day treatment partners, and give purpose to their lives. Through these accounts, next-of-kin vocalised the perceived rebellious behaviour of these men, and in the process of doing so neutralised their responsibility for the premature death of their relative.

## Introduction

While much attention is given to the experiences of men who struggle to engage with HIV services, less work has been done – and for very good reasons – to uncover the stories surrounding the most vulnerable group of men, namely those who die. With their death, their perspectives remain untold. However, HIV care is often a family affair, and next-of-kin can offer a close proxy for understanding the social circumstances surrounding the male relative’s death. For those reasons, we set out to explore next-of-kin perspectives on the circumstances that prevented their male relative from effectively engaging with HIV care service and their treatment. In doing so, we hope to heed the voices of next-of-kin who have experienced the slow demise of a relative due to HIV, and shed light on their role as ‘surrogates’ in understanding the experiences of people at the end of life.

Despite remarkable progress in availing HIV services to some of the poorest and hardest to reach populations in sub-Saharan Africa, mortality rates of people living with HIV (PLHIV) remain higher than those without HIV. Mortality rates are particularly high around the time of antiretroviral therapy (ART) initiation and amongst people who interrupt their antiretroviral treatment (Slaymaker et al., 2017). Men appear particularly disenfranchised when it comes to uptake of HIV testing services (Treves-Kagan et al., 2017; Venkatesh et al., 2011), treatment initiation (Gari, Martin-Hilber, Malungo, Musheke, & Merten, 2014; Hawkins et al., 2011), retention in care (Kranzer et al., 2010; Ochieng-Ooko et al., 2010) and AIDS-related mortality (Beckham et al., 2016; Druyts et al., 2013). Although some of this gender disparity may be attributed to a strong policy focus on pregnant women, enhancing women’s use of testing and treatment services (Dovel, Yeatman, Watkins, & Poulin, 2015), a number of commentators highlight the role of a so-called ‘masculinity factor’ (Nattrass, 2008) in heightening men’s disengagement with HIV services. Qualitative studies, for instance, have begun to unpick some of the pertinent male gender norms that shape men’s risk behaviours and engagement with HIV services. Accounts from a range of settings note how men’s interest to safeguard a reputation relating to their sexual prowess, physical strength and resilience can instill a fear and denial of HIV, preventing men from engaging with HIV testing and treatment services (Siu, Wight, & Seeley, 2014; Wyrod, 2008). Similar observations have been made in Zimbabwe (Skovdal et al., 2011), where local understandings of manhood (e.g. men being strong, in control, disease free, sexually promiscuous) were noted to often stand in stark contrast with nurses representations of ‘a good ART client’ (take instructions, accept HIV status, regularly hospital visits, refrain from extra-material sex and alcohol), discouraging some men from engaging with HIV services. This work supports the expanding body of work highlighting the influence of male gender norms on men’s health and health-seeking behaviours more generally (Galdas, Cheater, & Marshall, 2005; Noone & Stephens, 2008).

### Using next-of-kin as proxies for understanding life before death

The loss of a family member is an infrequent, emotional and important event, which is likely to heighten the memory of relatives. Addington-Hall and McPherson (2001) argue that this, coupled with the difficulty of interviewing people who are either too ill to participate in research, or not recognised as ‘dying’, makes next-of-kin suitable surrogates, even if the information generated is imperfect. In their review of after-death survey studies, comparing the after-death responses of relatives with those of patients in palliative care, they noted little agreement on questions pertaining to the presence or intensity of symptoms, but agreement on how health services were experienced [ibid.].

After-death interviews with next-of-kin in developing country settings tend to be categorised as either verbal autopsies (VAs) or social autopsies. VAs are done in settings with poor death registration systems and a need to determine or triangulate causes of death by capturing signs and symptoms observed by relatives prior to death (Byass et al., 2011). Social autopsies, in contrast, seek to unpick social and behavioural determinants of death, such as bottlenecks to care-seeking and illness management (Källander et al., 2011; Kalter, Salgado, Babille, Koffi, & Black, 2011). Social autopsies are commonly used to understand maternal, newborn and child mortality (Moyer, Johnson, Kaselitz, & Aborigo, 2017), but have also been used in conjunction with VAs in the context of HIV in sub-Saharan Africa (Njuki, Kimani, Obare, & Warren, 2014).

To understand the value of after-death interviews with next-of-kin, we need in-depth and contextual understandings of how next-of-kin react and respond to questions pertaining to the death of a relative (Gouda, Kelly-Hanku, Wilson, Maraga, & Riley, 2016). Our context – death on the HIV care cascade – warrants attention to the dissonances between biomedically and behaviourally rooted HIV services, which assume a level of individual autonomy and rational control, and the complex social realities of those affected by HIV (Seckinelgin, 2007; Skovdal et al., 2017; Wringe, Renju, Seeley, Moshabela, & Skovdal, 2017). Above we highlighted some of the factors that prevent men living with HIV from engaging optimally with HIV services. In the era of rolled out ART, there is only a short step from viewing men living with HIV and their carers as ‘autonomous and rational actors’ to representing them as ‘wholly responsible’ for the outcomes of their (in)actions and (dis)engagement with HIV services. As a consequence, when a person living with HIV dies, despite a history of contact with HIV services, their (in)actions, and those of their carers, may be put under the spotlight. This can have implications for how after-death interviews pan out. If next-of-kin feels evaluated in an after-death interview they may offer what Scott and Lyman (1968) call accounts, or statements to ‘explain unanticipated or untoward behaviour – whether that behavior is his own or that of others’. This, they argue, is a common ‘linguistic device employed whenever an action is subjected to valuative enquiry’ (p. 46).

Scott and Lyman (1968) outline different types of accounts, categorising them as either *excuses* or *justifications*. Excuses, they argue, are socially approved statements that relieve the interviewee from any form of responsibility. An interviewee may for example appeal to ‘biological drivers’, or human nature, such as sex and gender, to explain the behaviour of their male relative, or appeal to ‘defeasibility’, which relates to not being fully informed about what was going on. Justifications, on the other hand, relate to how actions that are generally considered impermissible, may on a particular occasion, given the context and circumstances, be permissible (Scott & Lyman, 1968). Here the interviewee may appeal to a ‘sad tale’, with past experiences explaining current behaviour, or ‘denial of victim’, where the interviewee may make the case that the deceased male relative deserved the fate thrust upon him. It is in this interactionist interview context that we heed the voices of next-of-kin and explore how they account for, or explain, the HIV-related behaviours and circumstances surrounding the death of their male relative.

## Methods

This article draws upon qualitative data from a larger multi-country study (the ‘bottlenecks study’) examining how PLHIV in sub-Saharan Africa, in the context of their social worlds, interact with HIV services (Wringe et al., 2017). Ethical approval was obtained from London School of Hygiene and Tropical Medicine (Ref: 10389) and by local ethics boards in the study locations. Informed and written consent was obtained from all participants.

### Settings and study participants

The study was conducted within seven rural health and demographic surveillance sites (HDSS) in Karonga (Malawi), Rakai and Kyamulibwa (Uganda), Kisesa (Tanzania), Kisumu (Kenya), Manicaland (Zimbabwe), and uMkhanyakude (South Africa). All sites are rural, poor and with generalised HIV epidemics. For people living in these communities, HIV has been part of the background of life for 30 years. Widespread messaging about HIV and treatment options, as well as personal experiences of knowing people living with, and dying from, HIV, form a backdrop to our participant’s understandings. To ascertain cause of death within the sites, VAs with head of households, as identified through HDSS surveys, were conducted by trained interviewers using a structured questionnaire, as per World Health Organization guidelines. Participants for this study were purposively sampled from VA data. Data managers from the respective sites compiled lists of deaths coded as caused by HIV-related illnesses or AIDS, which included the names of next-of-kin, their locations and contact details, if any. To avoid ‘outing’ the HIV status of a deceased person, only next-of-kin who had previously reported knowing the deceased’s HIV status in the VA interview were interviewed. To minimise recall bias, the relative should have passed away within the past four years. The deceased included individuals with a range of HIV care and treatment histories, including those who had enrolled in care, but not started ART, and those who had initiated ART.

In the ‘Bottleneck study’, we conducted 44 face-to-face interviews with next-of-kin across the seven sites. In this paper we draw on the 26 interviews that were conducted with next-of-kin of deceased male relatives (Table 1), giving detail to a collective story emerging from those interviews. The next-of-kin were typically female (62%), middle-aged and worked primarily in the informal economy or subsistence farming. Only a few were formally employed, with these generally holding low-skill and low-wage jobs, such as being a driver, a gas pump attendant or casual labourer in construction sites.

**Table 1.** Study participants.

HDSS		Karonga	Kisesa	Kisumu	Manicaland	Kyamulibwa	Rakai	uMkhanyakude	Total
Next-of-kin	Male	3	1	2	2	1	1	1	11
	Female	1	4	3	4	1	1	1	15
Total		4	5	5	6	2	2	2	26

### Data collection and analysis

The interviews took place in a convenient location of the participant’s choice, which was usually either at home or in private clinic areas. The interviews took place between October 2015 and April 2016 and were conducted in the local language by trained qualitative researchers originating from the area/site of the study. The researchers drew on a topic guide that examined the relationship between next-of-kin and the deceased, care sought or received by the deceased and the participant’s views on the underlying circumstances leading to their demise. In order to personalise each interview, the researchers consulted information previously provided in the VA tool. Interviews lasted 45–90 min and were digitally recorded, then anonymised, and either transcribed and translated (uMkhanyakude, Manicaland, Kisumu, Rakai, Kisesa, Karonga) or summarised into detailed field reports (Kyamulibwa).

Transcripts and summary reports were imported into computer-assisted qualitative data analysis software for interrogation and coding, led by MS and RS. A thematic network analysis (Attride-Stirling, 2001) identified latent and inductive themes, which were discussed with site coordinators. As different forms of accounts and masculine norms emerged from the thematic analysis, a second layer of more deductive coding – guided by accounts themes – resulted in the re-naming, merging or splitting up of existing codes and themes. Basic themes and common accounts appeals were clustered into three superordinate themes (Table 2). We came to these themes both on the basis of content, as well as our analytical interest to understand how masculine norms were manifested in the accounts. We found remarkable concordance across the seven sites in terms of the prominence of these three superordinate themes. The three themes form the structure of our findings. Quotes will be presented with identifiers specifying the relationship between next-of-kin and the deceased and the sub-Saharan African region from which they originate.

**Table 2.** Thematic network of emerging themes and common accounts appeals.

Basic themes	Next-of-kin accounts appeals	Superordinate themes	Global theme
Deceased male relative not disclosing HIV status to next-of-kin	Defeasibility	(i) The non-disclosing and un-supportable man	Next-of-kin emphasise the rebellious nature of their deceased male relative
Deceased male relative exhibits masculine norms	Gender norms		
Deceased male relative did not follow their advice	Defiance		
Deceased male relative may be mentally ill	‘Accidents’		
Deceased male relative adopted health-damaging behaviours	Health-damaging lifestyle	(ii) Care-free and rebellious lifestyle	
Deceased male relative not making an effort to survive	Self-infliction		
Deceased male relative not caring about his life	Capitulation		
Deceased male relative ran away	Avoidance	(iii) Unable to maintain caring relationships	
Deceased male relative abandons people who are trying to support	Self-infliction		

## Findings

### The non-disclosing and un-supportable man

Kin participants expressed frustration about the defiance of their male relative to disclose their HIV status and to be receptive to support, attributing this to shame, fear and a lack of self-acceptance of an HIV diagnosis. A mother of one deceased man provided a detailed account of how her son *defiantly* kept her in the dark. She said ‘I had advised him to go and get tested’, underlining that she did indeed try to help him and work out what his status was. Although she later learned that he had gone for testing and was in HIV care, this information only emerged when she found him very sick and had to take him to the local clinic.

It was only when he fell seriously ill and we took him to the clinic. That is when we were told that he was on the programme. I told them that I did not know but suspected it due to the constant illnesses that he had. (Mother of deceased, southern Africa)

Although she suspected his HIV positive status, she appealed to *defeasibility*, stressing she did not have the information or ability to act.

Aaaa I was troubled. I felt if it had been detected early he could have been assisted. I was troubled because I did not know what had happened, and could not protect my son. (Mother of deceased, southern Africa)

This particular mother not only explained her helplessness, but narrated how difficult and troublesome she found the whole experience of being kept in the dark, unable to support her son. The frustration of not being fully informed and ‘let in’ to the lives of their male relatives during sickness, was a common thread in the interviews.

While the mother above spoke about the defiance of her son to disclose his HIV status and accept care and support, a brother of another deceased man appealed to what Scott and Lyman (1968) call *accidents*, sudden and uncontrollable events (such as mental or physical illness). His sibling, despite taking a long time to accept his HIV positive status, eventually enrolled onto HIV care. However, his brother’s health quickly deteriorated when he decided to stop taking his medication, a turn of events his next-of-kin attributed to his poor mental state of mind.

He was not ready and had not accepted it well. I think he had mental problems and thought that since he was taking tablets and was feeling well, he might have thought that he had been cured. This is just my thoughts. (Brother of deceased, southern Africa)

Another participant experienced a similar problem with his brother not coming to terms with his possible HIV status. In his account, explaining his brother’s lack of engagement with HIV services, he appealed explicitly to *gender norms*, suggesting that ‘men are like that’.

He thought that it was not good to be seen by people that he was sick […] He feared testing positive, and he thought it was me who had it. You know men are like that, they think that it is you [everyone else] who got it. (Brother of deceased, southern Africa)

Care-free and rebellious lifestyle

Some kin spoke about how their relative ascribed to a care-free and rebellious lifestyle, deliberately rejecting the patient-persona. A mother illustrates this well by giving an account of how her son, ever since he was a child, engaged in behaviours she deemed ‘mischievous’. When accounting for his premature death, she appealed to a tenacious *health-damaging lifestyle* of drinking alcohol and smoking, a lifestyle she believed got him sick in the first place, despite having been told to stop by the health worker.

When he went to the clinic he was told that he was found with the virus. He was told to stop drinking beer and smoking, but didn’t. As a child he was being mischievous and started drinking beer and smoking. This is when he started to be sick. (Mother of deceased, southern Africa)

A similar tale of defying the health worker’s advice to live a healthy lifestyle, free from drinking alcohol and smoking, emerged from a younger brother whose older brother deliberately tried to deceive him.

Dr. BM also told him to stop drinking alcohol and smoking because they would affect his health further. He did as directed for four months and went back to his old habits. Thereafter, he developed a pill burden. He reached a point and did not want to take them any further. He used to wake up early and got the pills from the container to pretend that he had swallowed them. (Brother of deceased, eastern Africa)

A number of next-of-kin provided accounts of male relatives defying instructions by healthcare workers. Some of these accounts appealed to *self-infliction*, meaning they blamed the relative for his own premature death. Some next-of-kin felt that their male relative did not try hard enough to survive, or simply did not care enough about their life to engage with HIV treatment services seriously.

If you go to the clinic when you are sick you should do what you can to survive. If you are not trying to survive, you will not succeed. (Mother of deceased, eastern Africa)

I can say that he did not care. He did not care about his life which he took simply as something not difficult. We could see that there were elements that he did not care. (Brother of deceased, southern Africa)

Stressing the perceived responsibility of the individual, and not health services, a mother indirectly blamed her son for not engaging with the HIV services.

We cannot blame the government because those who follow instructions and take these drugs are living healthy. (Mother of deceased, eastern Africa)

It is difficult to speculate on the roots of this laissez-faire attitude of their male relatives based on the accounts, but some next-of-kin talked about how acquiring HIV may have caused them to lose their will to live, appealing to a *capitulation*. An aunt, of a man who had initially responded well to treatment, speculated that her nephew’s sudden interest to drink excessively was, in fact, a suicide attempt.

When he started treatment, he improved because of adherence. Then he started to drink beer. I do not know, maybe he did it because he wanted to die? I do not know what he was thinking because he was now drinking too much. (Aunt of deceased, southern Africa)

A mother of a deceased experienced something similar. Her son suddenly lost hope, and decided to stop taking his pills.

He was not taking them [antiretroviral drugs]  …  if you give him he will just spit them out. He had lost hope  …  even his wife could not manage to give him. He did not want the drugs. (Mother of deceased, eastern Africa)

She tried to reason with him, referring to his quiet nature: ‘he was a soft man who was never talking … he was never talking’ but to no avail.

### Unable to maintain caring relationships

Some next-of-kin described their male relatives as having weak family ties, and as unable to maintain caring relationships that would avail day-to-day treatment partners and support. Deceased relatives who had migrated for work and established a family in their new location were often described as having weak links to their family, making it difficult for them to return ‘home’ for care if they were abandoned by their wives. Other deceased relatives were said to have run away from their home area and close kin when learning about their HIV positive status. The niece of a deceased relative in eastern Africa said: ‘he ran away after he had gone to take the test’. This running away was often attributed to shame. A number of next-of-kin appealed to *avoidance* in their accounts, alluding to how male relatives deliberately avoided contact with them. Two mothers spoke about their son’s disappearances, and the unpredictable, sporadic and limited contact they had with their sons, never knowing whether they were dead or alive.

We would just see him coming home and we would be surprised that he was still alive. We would fear that he was no longer alive because he spent a long time away from us. (Mother of deceased, southern Africa)

He would refuse drinking medicine … He started complaining, ‘I am always drinking medicine but I don’t get cured … .’Then he ran away, maybe to his father […] Suddenly he ran away again […]. Suddenly I received information that he had died. (Mother of deceased, southern Africa)

The deceased relatives’ HIV positive status was said to take its toll on their relationships. Next-of-kin gave examples of their deceased male relatives who were abandoned by their wives when they were found to be HIV positive. Others gave examples of couples who separated long after finding out they were positive. In an interview with a brother of a deceased man, he blamed his deceased brother for not making his marriage work, as it, and the practical support his wife offered, was what he believed, had kept his brother alive.

The wife supported him to go to the clinic and take the treatment whilst they were still together. When they separated that is when he stopped taking the treatment. If a person abandons something that is helping you, shows that person does not care about it. (Brother of deceased, southern Africa)

## Discussion

We set out to foreground the voices of next-of-kin who have witnessed the demise of a male relative due to HIV, in the hope of generating insight into the lives of some of the hardest to reach men in the HIV response. We found that next-of-kin in the after-death interviews felt a need to explain and justify their (in)actions, and offered detailed accounts of their perspectives on the circumstances surrounding the death of their male relative. From an interactionist perspective, our interview situation appeared to unintentionally subject the participants to a perceived moral judgment, which they responded to in the interviews. In the process of doing so, they represented the men as rebellious and impossible to support, relinquishing any form of responsibility for their premature death.

Echoing qualitative observations around the ‘masculinity factor’ (Nattrass, 2008) in men’s disengagement with HIV services, next-of-kin spoke about how their deceased male relative delayed care-seeking and refrained from disclosing their HIV status – avoiding the reality of being HIV positive. Similar observations have been made in South Africa, where men were observed to adopt various strategies to avoid or postpone disclosing their HIV status (Chikovore et al., 2016). In our study, next-of-kin expressed that common avoidance strategies of the deceased male relative were to either not disclose their HIV status or to run away, making it difficult for next-of-kin to take on a care role. Our findings chime with those from Zimbabwe (Skovdal et al., 2011) and Uganda (Mburu et al., 2014) where participants also noted how their male relative did not want to accept the patient-persona, deliberately ignoring instructions received by the health worker, and instead turned to harmful behaviours, such as drinking alcohol, as a way perhaps to ‘normalise’ or ‘end’ life. A number of studies in sub-Saharan Africa have highlighted how alcohol use is linked to men’s concern about demonstrating masculinity (Brown, Sorrell, & Raffaelli, 2005), and the implications of this for HIV-related behaviour (Nkosi, Rich, Kekwaletswe, & Morojele, 2016; Rich, Nkosi, & Morojele, 2015; Woolf-King & Maisto, 2011). This study supports the ample literature that highlights how masculine norms come to shape men’s engagement with HIV risk, prevention methods and treatment services. While it is evident that men’s concern about demonstrating masculinity can have a detrimental impact on their health, well-being, and life, this study also shows how people around them, their next-of-kin, are affected. The care ethics (cf. Tronto, 1993) of next-of-kin were challenged by the men’s unwillingness to receive care, much to their frustration.

Many of the findings discussed above emerged through next-of-kin’s accounts, or explanations, for the (in)actions of their deceased male relative, as well their own scope to act and care. They appealed to the defiance of their male relative to disclose their HIV status and accept care from next-of-kin, their lack of will to try and survive, their mischievous behaviour, and troubled upbringing. Some made direct appeals to their gender and sex, while others thought their death was self-inflicted if unable to maintain caring relationships that would avail day-to-day treatment partners, and give purpose to their lives. Through these accounts, next-of-kin painted a picture of their deceased male relatives as rebellious and care-free. Our findings about how masculine norms shape men’s engagement with HIV services must be considered in light of this context. In representing their deceased relative as rebellious, next-of-kin created a distance between the actions of their relative and what they, as next-of-kin, could have done to help, and simultaneously neutralised their responsibility for the premature death of their kin. Our next-of-kin participants effectively used the interview situation to label their deceased male relative as ‘rebellious’ and ‘care-free’, in order to relinquish any essence of responsibility and control over the premature death of their male relative. This is further supported by the absence of structural explanations for their male relative’s behaviour. In a seminal paper, Courtenay (2000) argues that social and institutional structures play a central role in shaping the reasons why men behave in ways that adhere to certain versions of masculinity. A recent study from Zimbabwe highlights the power of social and structural forces in determining how different masculine norms come to shape HIV risk behaviours and engagement with HIV services (Rhead et al., 2019). Rhead and colleagues noted how men’s participation in social groups amplifies their masculine gender norms, whilst area of residence, material status, level of education and church denomination all had varying effects on different male gender norms, which in turn were associated with different levels of engagement with HIV services. Moreover, next-of-kin made little reference to the shortcomings in the delivery of health services, despite amble research highlighting this an important role in people’s decision to interrupt treatment or not initiate ART (Beckmann, 2013; Doyal, 2016; Mattes, 2011).

Our study has several strengths and limitations. Sampling and recruitment were aided by HDSS databases. Experienced research teams, with ample experience of working together in cross-country studies, facilitated the coordination of data production and analysis. One limitation of the study was that we did not achieve saturation in every site as we were limited in the number of interviewees that were eligible, could be identified, and agreed to participate, in each site. After-death interviews, verbal and social autopsies are ordinarily carried out with a relative. A limitation of this is that the relative may be bereaved and emotionally vulnerable, or experiencing the consequences for themselves of the man’s death (e.g. loss of income) – all of which can shape next-of-kin’s perspectives. Future research could consider conducting after-death interviews with more than one relative, nurses, friends or members of a support group, if applicable, as well as participant observations to triangulate and nuance the circumstances surrounding the death of this group of men. Participant observations would also help to unpack the broader social and structural characteristics that shaped the inclination of next-of-kin to emphasise their deceased male relatives’ rebellion to widespread notions of positive living.

It is evident that our next-of-kin participants had thought about whether they could have done more to prevent the death of their male relative, and in the process have rationalised their premature death, perhaps as a way to cope with their self-perceived responsibility. Our findings highlight the urgent need to make sure relatives are integrated into current health systems, and form part of the two-way ‘contract’ that exists between the health system and PLHIV (Kulzer et al., 2012; van Rooyen et al., 2016). More often than not, next-of-kin are peripheral to formal care – often left hanging with little information or control to offer care, which is incongruent with traditional caregiving structures. It is noteworthy that the concept of ‘treatment supporter’ only featured indistinctively in the interviews – a sign that current policy, which tries to bring in community and family support, is not always effective at garnering support for those that need it the most. Our findings noted that many of the deceased men, due to the shame they felt, were unable to seek support from their relatives. Some men living with HIV find it easier to seek support from non-relatives, such as post-test clubs, AIDS support groups, work-colleagues or friends (Berg et al., 2004; Campbell et al., 2011; Kalichman, DiMarco, Austin, Luke, & DiFonzo, 2003). None of our next-of-kin participants alluded to the availability of such support, suggesting that such alternative forms of support are not accessible to all men.

Our findings also open up a discussion on the value of after-death interviews. Whilst we cannot conclude that the information provided by next-of-kin mirror the experiences of their male relatives, the challenges that they identified did resonate with much of what has been written about the role of masculinity in shaping engagement with HIV services. Even if the information is imperfect, it is likely to be the closest we will get to understanding the circumstances surrounding their death. Moreover, according to Addington-Hall and McPherson, the views of next-of-kin carry ‘their own validity even if they do not tally exactly with patients’ experiences, as it is their memory of events which lives on, and which may impact on their adjustment to bereavement and subsequent health’ (Addington-Hall & McPherson, 2001, p. 789). Whilst we did not follow any of the frameworks that are generally used to guide social autopsies, our approach of allowing next-of-kin to talk more broadly about the circumstances of their relative’s death, bear a resemblance to social autopsies. This approach, coupled with our interactionist interview context, facilitated a distancing of responsibility, which made it easier for them to cope with, and talk about, the death of their relative. Given the insights we have learned, there is a need to either capture the social narratives in VAs more fully, by training interviewers to prompt and record social and behavioural determinants during the initial narrative questioning, or by conducting separate in-depth social autopsies, akin to what we have done, with a smaller sample.

## Data Availability

The data that support the findings of this study are available from Dr Alison Wringe, upon reasonable request.
